# Osteoid osteoma of the thumb: A case report

**DOI:** 10.1016/j.ijscr.2025.111540

**Published:** 2025-06-20

**Authors:** Abdullah Almohimeed, Mohamed Achraf Ferjani, Mohamed Ali Bekkay, Khaled Kamoun

**Affiliations:** a HafrAl-Baten Health Cluster, Hafar Al Batin, Saudi Arabia; b University of Tunis El Manar Faculty of Medicine of Tunis, Tunis, Tunisia

**Keywords:** Adolescent, Proximal phalanx, Hand tumors, Surgical resection, Case report

## Abstract

**Introduction and importance:**

Osteoid osteoma (OO) is a benign bone-forming tumor that rarely affects the small bones of the hand, particularly the thumb. Its diagnosis in this location can be challenging due to its atypical presentation and the potential for mimicking other conditions.

**Case presentation:**

We present the case of a 16-year-old female who presented with persistent, painful swelling of the proximal phalanx of her thumb. There was no history of trauma or infection. Radiographs revealed sclerosis and a hollowed-out lesion in the distal aspect of the phalanx, raising suspicion for chronic osteomyelitis. This initial misdiagnosis contributed to a delay in definitive treatment. Surgical en bloc resection was performed using a mid-axial approach under C-arm guidance. Intraoperative cultures were negative for microorganisms, and histopathological examination confirmed the diagnosis of OO.

**Clinical discussion:**

OO of the thumb is extremely rare and can be misdiagnosed due to its resemblance to infectious or inflammatory conditions. Delayed diagnosis may lead to prolonged pain and functional impairment. Imaging, particularly CT scans, plays a crucial role in identifying the nidus characteristic of OO. Surgical excision remains the gold standard for treatment, providing definitive relief and preventing recurrence.

**Conclusion:**

This case highlights the importance of considering OO in the differential diagnosis of persistent thumb pain and swelling, even in the absence of trauma or infection. Surgical resection provided an excellent outcome in this case, emphasizing the effectiveness of this treatment modality for OO in this unusual location.

## Highlights

• Rare case of osteoid osteoma in the proximal phalanx of the thumb in an adolescent.
• Atypical presentation mimicked infection, leading to an initial misdiagnosis and delayed treatment.
• En bloc resection was performed successfully, ensuring complete removal and symptom resolution.
• Histopathological analysis confirmed diagnosis, ruling out other benign or malignant bone lesions.
• Excellent long-term outcome with full recovery and no recurrence after five-year follow-up

## 1 Introduction

Osteoid osteoma (OO) is a relatively common benign osteoblastic bone tumor, typically arising in the first three decades of life [1,2]. It accounts for approximately 10–12 % of all benign bone tumors and is characterized by a well-demarcated nidus of osteoid and woven bone, often surrounded by a zone of reactive sclerosis [3]. While OO most frequently occurs in the long bones of the lower extremities, particularly the femur and tibia [1], it can affect any bone in the body. Involvement of the small bones of the hand is relatively uncommon, accounting for only 5–15 % of cases [4,5]. Within the hand, the proximal phalanx is the most commonly reported site, followed by the metacarpals [5,6]. Lesions in the thumb are less frequent [7], and involvement of the distal phalanx is exceedingly rare [8,9].

Due to its infrequent occurrence and often atypical clinical and radiological presentation, the diagnosis of OO in the thumb can be challenging. The symptoms can mimic other conditions, such as tenosynovitis, infection, or other benign tumors, leading to delays in diagnosis and potentially inappropriate treatment [10,11]. This diagnostic difficulty is further compounded in cases involving the proximal phalanx, as there is less available literature describing the characteristic features of OO in this location.

This case report presents an unusual case of OO involving the proximal phalanx of the thumb in a 16-year-old female. We aim to describe the clinical and radiological presentation, diagnostic process, surgical management, and outcome of this rare case. By highlighting this unusual presentation, we emphasize the importance of including OO in the differential diagnosis for patients presenting with persistent thumb pain and swelling, particularly adolescents. The work has been reported in line with the SCARE criteria [12].

## 2 Case report

### 2.1 Patient and observation

#### 2.1.1 Patient information

A 16-year-old right-hand-dominant female presented with a three-month history of progressively worsening pain with a Visual Analog Scale (VAS) score of 3 to 5 and swelling in her left thumb. The pain was described as dull and aching, worse at night and partially relieved by over-the-counter ibuprofen. There was no history of trauma, infection, or other preceding events related to the onset of symptoms. Her medical history was unremarkable, with no known chronic illnesses, bleeding disorders, or allergies. Family history was non-contributory for musculoskeletal conditions or genetic disorders. The patient was a non-smoker and reported no significant psychosocial stressors. Prior to presentation, she had attempted conservative management with rest, ice, and over-the-counter pain medication, but experienced minimal relief of her symptoms. No other interventions had been attempted.

#### 2.1.2 Clinical findings

On physical examination, the patient's left thumb exhibited mild swelling and erythema localized to the proximal phalanx. Palpation elicited tenderness specifically over the distal aspect of the proximal phalanx, with no palpable mass or crepitus. The range of motion of the interphalangeal and metacarpophalangeal joints was full and pain-free; however, active and passive range of motion at the first carpometacarpal joint reproduced the patient's pain, particularly with abduction and opposition. There was no appreciable warmth or tenderness over the thenar eminence or wrist joint. Neurovascular examination of the left hand revealed intact sensation, capillary refill, and radial and ulnar pulses. Strength testing was 5/5 in all muscle groups of the hand and wrist compared to the contralateral side. No regional lymphadenopathy was noted. The patient's fingernails were normal, with no clubbing, ridging, or discoloration observed.

#### 2.1.3 Timeline of current episode

Three months prior to presentation, the patient experienced an insidious onset of dull, aching pain in her left thumb, initially mild and intermittent. The pain progressively worsened over the next two months, becoming more constant and disturbing sleep. She began using over-the-counter ibuprofen, which provided partial relief. One month prior to presentation, swelling and mild redness developed in the affected thumb, and the pain continued to escalate despite regular ibuprofen use. On the day of presentation to the emergency department, she sought further evaluation and pain management due to persistent pain, swelling, and erythema. She was subsequently referred to an orthopedic specialist, and imaging studies (X-ray) were performed during a clinic visit. En bloc resection of the lesion was performed. Postoperative follow-up visits revealed uneventful healing, progressive improvement in range of motion and pain levels, and ultimately a full return to normal activities with no evidence of recurrence.

#### 2.1.4 Diagnostic assessment

**Fig. 1:**
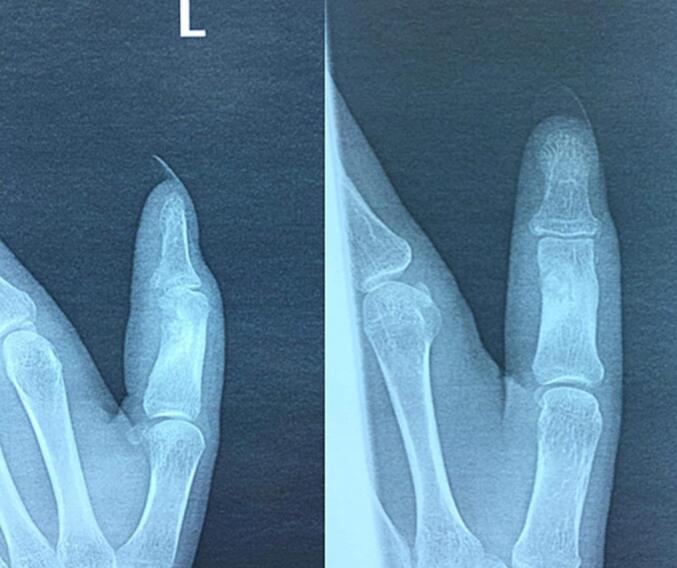
Sclerotic hollow lesion in distal proximal phalanx of left thumb on radiographs.

Initial evaluation included a physical exam, which revealed the findings described previously. Standard radiographs of the left thumb demonstrated a sclerotic, hollowed-out lesion in the distal aspect of the proximal phalanx (Fig. 1).

Laboratory testing, including a complete blood count, erythrocyte sedimentation rate, and C-reactive protein, was within normal limits, further supporting the diagnosis of OO and lessening the likelihood of an infectious or inflammatory process.

#### 2.1.5 Diagnosis

Based on the clinical presentation, imaging findings, and normal laboratory results, a presumptive diagnosis of OO of the left thumb proximal phalanx was made. Other diagnoses considered included chronic osteomyelitis, enchondroma, and other benign bone-forming tumors. However, the characteristic appearance of the lesion, along with the absence of inflammatory markers and the patient's response to NSAIDs, strongly favored the diagnosis of OO. Histopathological examination of the resected surgical specimen confirmed the final diagnosis of osteoid osteoma. As OO is a benign lesion with no metastatic potential, the prognosis is excellent following complete surgical excision. Recurrence is possible but uncommon after en bloc resection.

#### 2.1.6 Therapeutic interventions

**Fig. 2:**
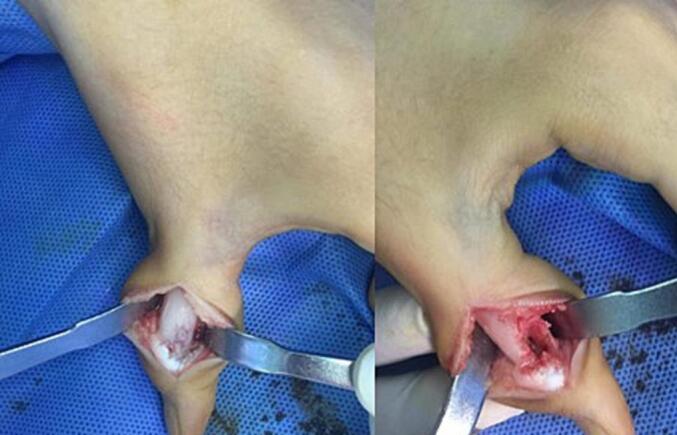
En bloc resection of the tumor in the first phalanx of the thumb.

The patient underwent en bloc resection of the lesion under C-arm guidance, through a lateral incision on the left thumb, performed under regional block anesthesia supplemented with local infiltration. Intraoperatively, a well-circumscribed lesion consistent with an osteoid osteoma was identified and excised (Fig. 2).

**Fig. 3:**
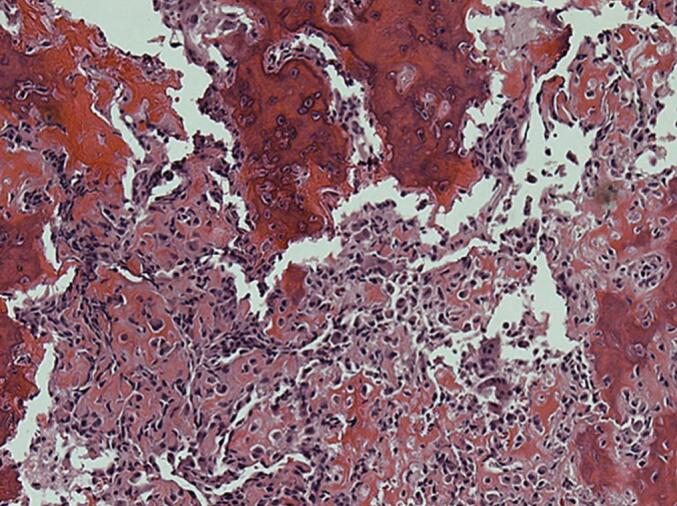
Histologic Features of Osteoid Osteoma: Central Nidus with Woven Bone Trabeculae and Osteoblast Lining.

Careful dissection was performed to preserve surrounding neurovascular structures and minimize damage to the adjacent joint. The resected specimen was sent for histopathological examination (Fig. 3).

Postoperatively, the patient received oral as needed for pain management. The initial postoperative dressing consisted of a sterile bandage and a thumb spica splint. The splint was removed after one week, and the patient began gentle range-of-motion exercises under the guidance of a hand therapist, followed by continued physiotherapy for a total of three months.

#### 2.1.7 Follow-up and outcome of interventions

At the most recent follow-up, 5 years postoperatively, the patient remained asymptomatic, with no pain, swelling, or limitation of motion. Radiographic imaging confirmed bony healing at the surgical site with no evidence of recurrence. The patient reported excellent satisfaction with the outcome and experienced no adverse or unanticipated events throughout the follow-up period.

#### 2.1.8 Patient perspective

“My thumb pain was progressively worsening, impacting sleep and daily activities. Post-surgery, the pain resolved quickly, and I regained full, pain-free function and am very satisfied.”

## 3 Discussion

Osteoid osteoma (OO) is a relatively common benign bone tumor, but its occurrence in the small bones of the hand, particularly the thumb, is rare [1,2]. This unusual location presents diagnostic challenges, as the clinical and radiological findings can mimic other conditions, such as chronic osteomyelitis, tenosynovitis, or other benign tumors [3,4]. The rarity of OO in the thumb, coupled with its potential for atypical presentation, often leads to delays in diagnosis and treatment [5]. As our case demonstrates, even with classic features of OO, such as nocturnal pain and some relief with NSAIDs, the unusual location can lead to initial misdiagnosis. Our patient's initial radiographs were interpreted as showing a sclerotic lesion suggestive of chronic osteomyelitis, highlighting the importance of considering OO in the differential diagnosis for persistent thumb pain.

While plain radiographs can sometimes reveal the characteristic nidus and surrounding sclerosis, advanced imaging, such as computed tomography (CT), is often crucial for accurate diagnosis and surgical planning [6]. In our case, the absence of inflammatory markers and a normal white blood cell count helped to rule out infection. The decision to perform an en bloc resection, rather than curettage or radiofrequency ablation, was based on the lesion's location and size, and the desire to minimize the risk of recurrence [7,8]. The lateral approach provided good access to the lesion while minimizing disruption of surrounding structures.

The excellent long-term outcome observed in our patient, with complete resolution of pain and restoration of function, is consistent with the expected prognosis for OO following complete surgical excision [9,11]. The five-year follow-up confirmed the absence of recurrence, further validating the effectiveness of en bloc resection in this case.

This case report underscores the importance of maintaining a high index of suspicion for OO in patients presenting with persistent thumb pain, even in the absence of trauma or a clear history of infection. Advanced imaging, particularly CT, plays a vital role in establishing the diagnosis and guiding appropriate surgical management. En bloc resection offers a reliable treatment option with an excellent prognosis for long-term pain relief and functional recovery.

## 4 Conclusion

This case report emphasizes the diagnostic challenges associated with osteoid osteoma (OO) in unusual locations, such as the proximal phalanx of the thumb. Even with suggestive clinical features, the atypical presentation can mimic other conditions, leading to initial misdiagnosis. Advanced imaging, particularly CT, is crucial for accurate diagnosis and surgical planning. En bloc resection, as performed in this case, offers a reliable treatment option with an excellent long-term prognosis for pain relief and functional recovery in OO of the thumb. This case contributes to the limited literature on OO in this specific location and reinforces the importance of considering this diagnosis in adolescents presenting with persistent thumb pain and swelling.

## Author contribution

Patients management: Kamoun Khaled and Mohamed Ali Bekkay. Data collection: Mohamed Achraf Ferjani. Manuscript drafting: Mohamed Achraf Ferjani. Manuscript revision: Almohimeed Abdullah and Khaled Kamoun. All the authors read and approved the final version of this manuscript.

## Consent to publish

Written informed consent was obtained from the patient's parents/legal guardian for publication and any accompanying images. A copy of the written consent is available for review by the Editor-in-Chief of this journal on request.

## Ethical approval

Ethics approval is not required for case reports in our institution as they are deemed not to be research. This exemption is in accordance with the policies of Hafar El Baten Health Cluster.

## Guarantor

Mohamed Achraf Ferjani.

## Research registration number

It's a case report of one case and retrospective case.

No section for case report retrospectively study was found in the register provided.

## Funding

This research did not receive any specific grant from funding agencies in the public, commercial, or not-for-profit sectors.

## Conflict of interest statement

We declare that there are no financial, professional, or personal conflicts of interest related to this manuscript.

## Data Availability

Underlying data. No data are associated with this article.
